# Investigation on potential associations of oxidatively generated DNA/RNA damage with lung, colorectal, breast, prostate and total cancer incidence

**DOI:** 10.1038/s41598-019-42596-x

**Published:** 2019-05-08

**Authors:** Xīn Gào, Bernd Holleczek, Katarina Cuk, Yan Zhang, Ankita Anusruti, Yang Xuan, Yiwei Xu, Hermann Brenner, Ben Schöttker

**Affiliations:** 1 Division of Clinical Epidemiology and Ageing Research, German Cancer Research Center (DKFZ), D-69120 Heidelberg, Germany; 2 Network Aging Research, Heidelberg University, D-69115 Heidelberg, Germany; 3 Division of Preventive Oncology, German Cancer Research Center (DKFZ) and National Center for Tumor Diseases (NCT), D-69120 Heidelberg, Germany; 4 Saarland Cancer Registry, D-66119 Saarbrücken, Germany; 5 German Cancer Consortium (DKTK), German Cancer Research Center (DKFZ), D-69120 Heidelberg, Germany; 6 Department of Surgery, School of Medicine and Public Health, University of Wisconsin, Madison, WI 53792 USA; 7 Institute of Health Care and Social Sciences, FOM University, D-45127 Essen, Germany

**Keywords:** Predictive markers, Cancer, Colorectal cancer, Risk factors

## Abstract

Oxidative stress has been linked to cancer development in previous studies. However, the association between pre-diagnostic oxidatively generated DNA/RNA damage levels and incident cancer has rarely been investigated. Urinary oxidized guanine/guanosine (OxGua) concentrations, including 8-hydroxy-2′-deoxyguanosine, were assessed in 8,793 older adults in a population-based German cohort. 1,540 incident cancer cases, including 207 lung, 196 colorectal, 218 breast and 245 prostate cancer cases were diagnosed during over 14 years of follow-up. Associations of OxGua levels with cancer outcomes were not observed in the total population in multi-variable adjusted Cox regression models. However, in subgroup analyses, colorectal cancer incidence increased by 8%, 9% and 8% with one standard deviation increase in OxGua levels among current non-smokers, female and non-obese participants, respectively. Additionally, among non-smokers, overall and prostate cancer incidences statistically significantly increased by 5% and 13% per 1 standard deviation increase in OxGua levels, respectively. In contrast, OxGua levels were inversely associated with the risk of prostate cancer among current smokers. However, none of the subgroup analyses had p-values below a threshold for statistical significance after correction for multiple testing. Thus, results need to be validated in further studies. There might be a pattern that oxidatively generated DNA/RNA damage is a weak cancer risk factor in the absence of other strong risk factors, such as smoking, obesity and male sex.

## Introduction

The term “oxidative stress (OS)” refers to an imbalance in which the production of reactive oxygen species (ROS) overwhelms the capacity of antioxidant defense systems leading to a dysregulation of redox signaling and/or damage to biomolecules^1^. OS has long been known to be involved in cancer. Excessive levels of ROS may directly react with nucleic acids leading to mitochondrial and nucleus genomic instability, which facilitates carcinogenesis^2^. ROS may also activate or inhibit downstream signaling pathways promoting cancer development^2–6^. In addition, OS can contribute to carcinogenesis through epigenetic mechanisms^7–10^. For instance, the patterns of DNA methylation can be affected by oxidative DNA damage causing aberrant gene expression^9^. However, studies with humans on associations of ROS with cancer risk are not possible due to the short half-life of ROS in human specimens. Therefore, OS related biomarkers are needed for epidemiological studies as proxies for the effect of OS on cellular molecules^11,12^.

The Oxidized guanine/guanosine (OxGua) molecules, including 8-hydroxyguanine (8-OHGua) and its nucleoside forms 8-hydroxy-2′-deoxyguanosine (8-OHdGuo) and 8-hydroxyguanosine (8-OHGuo), have been used as biomarkers to assess the intensity of ROS-induced DNA damage in epidemiological studies. These molecules are formed from the attack of hydroxyl radicals on the guanine of the DNA strand and RNA strand^12^. Unrepaired DNA lesion can lead to GC → TA transversion mutations. However, the DNA lesion can be corrected by the base excision repair process, which is initiated by 8-oxoguanine glycosylase (OGG1). 8-OHGua is removed by OGG1 and the formed Apurinic/apyrimidinic site can be cleaved by an endonuclease^13^. The RNA is more vulnerable to oxidative stress due to its single-stranded structure. 8-OHGuo is one of 20 types of RNA damage and might lead to synthesis of anomalous proteins^14^. Degradation of oxidized RNA stands is mainly performed by ribonucleases^14^. Ultimately, the OxGua degradation products of oxidatively damaged DNA and RNA are being secreted into urine. The OxGua molecules are very stable in frozen urine samples (shown for up to 15 years of storage^15^). Therefore, OxGua is a biomarker of OS that can be measured in stored baseline urine samples from studies with long-term follow-up. However, due to inconsistent findings from prospective observational studies, it is unclear whether urinary OxGua levels are associated with cancer risk^15–20^.

In this study, OxGua levels were measured to determine the prospective association with total cancer incidence and the incidences of the most common site-specific cancers (i.e., lung, colorectal, breast and prostate cancer) in a population-based cohort study with 14 years of cancer follow-up.

## Methods

### Study population

This investigation is based on the ESTHER study (German Name: “Epidemiologische Studie zu Chancen der Verhütung, Früherkennung und optimierten Therapie chronischer Erkrankungen in der älteren Bevölkerung”), an ongoing population-based cohort. The study design has been reported elsewhere in detail^21,22^. Briefly, the cohort was initiated during 2000 and 2002 in Saarland, a federal state in southwest Germany. At baseline, 9,940 subjects, aged 50–75 years, were recruited by their general practitioners during a general health check-up. In current study, 783 subjects with a history of any cancer except non-melanoma skin cancer were excluded. Furthermore, we excluded 168 individuals with missing urine samples, and 196 study participants for whom urinary OxGua levels could not be measured, resulting in an analytical sample of 8,793 individuals. Analyses on breast and prostate cancer were restricted to females (n = 4,853) and males (n = 3,940) study participants, respectively.

### Ethical approval and informed consent

This study was approved by the ethics committees of the University of Heidelberg and the state medical board of Saarland. Personal information and human specimens were collected after obtaining signed and informed written consent from all study participants. The study was conducted in accordance with the Declaration of Helsinki.

### Laboratory analyses

At baseline, blood and spot urine samples were collected during the health check-up and shipped to the study center using a temperature-controlled supply chain. After arrival in our lab, we stored the urine samples at −80 °C until further processing. Urinary OxGua concentrations were assessed with the DNA/RNA Oxidative Damage ELISA Kit of Cayman (Ann Arbor, Michigan, USA), which detects all three OxGua species: 8-OHGua from either DNA or RNA, 8-OHdGuo from DNA, and 8-OHGuo from RNA. Accordingly, this assay captures a more complete set of biologically relevant products of oxidatively generated DNA/RNA damage than 8-OHGua specific assays. The dilution factor was 800-fold. Urinary creatinine was determined by the kinetic Jaffe method for renal function adjustment of spot urine samples and OxGua levels are being reported in the unit “μg/g creatinine”. Serum C-reactive protein (CRP) and total cholesterol levels were measured by immunoturbidimetry and an enzymatic colorimetric assay, respectively.

### Outcome ascertainment

Up to the end of 2014, all cancers were recorded by linking study participants to the Saarland Cancer Registry. The 10^th^ Revision of the International Statistical Classification of Diseases (ICD-10) was used for coding of the cancer sites. Total cancer incidence included all cancer sites except non-melanoma skin cancers (ICD-10 code C44). Lung, colorectal, breast and prostate cancer were coded as C34, C18-C21, C50 and C61, respectively.

### Covariates

Sociodemographic characteristics, lifestyle and dietary factors were collected by a standardized self-administered questionnaire. Self-reported smoking information was confirmed to be reliable in a subgroup of 1,500 study participants with serum cotinine measurements^23^. Height and weight were measured by general practitioners during the health check-up and documented on a standardized form.

### Statistical analyses

Statistical Analysis System (SAS, version 9.4, Cary, North Carolina, USA) was used to perform all statistical analyses. Statistical tests were two-sided using a significance level of 0.05.

Variations in urinary OxGua levels according to established cancer risk factors were assessed by comparing proportions (by Chi-square tests) or medians/means (by Wilcoxon-Mann-Whitney tests) in OxGua level tertiles while using the bottom tertile as the reference.

Cox proportional hazards models were performed to estimate hazard ratios (HRs) with corresponding 95% confidence intervals (95% CIs) to investigate the associations of OxGua levels with the incidence of total cancer and four common cancers. Death is a competing risk of the cancer of interest during the follow-up. Therefore, competing risk of mortality modelling was applied in Cox regression models (except the mortality due to the cancer of interest). The main model, in which OxGua levels were categorized in tertiles as well as modelled linearly, was adjusted for potential confounders, including age, sex, physical activity, body mass index, smoking status, alcohol consumption and dietary factors (fruit, vegetable and red meat consumption). These variables were modelled as displayed in Table 1. In the main analyses, smoking status was modelled in the categories shown in Table 1 and in sensitivity analyses, the continuous variables “current tobacco consumption in grams per day” and “pack-years of smoking” were used. In addition, subgroup analyses with stratification by age (50–64/65–74 years), sex, smoking status (current smoking/non-smoking) and BMI (<30/≥30 kg/m^2^) were performed. In a further sensitivity analysis cancers diagnosed in the first 2 years of follow-up were excluded to address potential reverse causality bias by early events. Furthermore, in order to correct for multiple testing in subgroup analyses, both the false discovery rate (FDR; using Proc Multtest (SAS 9.4)) and the more conservative Bonferroni method were applied.

**Table 1:** Baseline characteristics of the study population, The ESTHER Study (2000–2016).

Characteristics	*n* ^a^	Measure	Values
Age (years)	8793	Median (IQR)	62 (57–67)
Sex	8793	–	–
Female	4853	%	55.2
Education (years)	8568	–	–
<9	6395	%	74.6
9–11	1229	%	14.4
≥12	944	%	11.0
Smoking status	8520	–	–
Never smoker	4289	%	50.3
Former smoker, quitted >20 years ago	1253	%	14.7
Former smoker, quitted 5–≤20 years ago	1157	%	13.6
Former smoker, quitted 0–≤5 years ago	383	%	4.5
Current smoker, 0–≤15 g tobacco/day	620	%	7.3
Current smoker, 15–≤30 g tobacco/day	716	%	8.4
Current smoker, >30 g tobacco/day	102	%	1.2
Alcohol consumption (g/day)^b^	7923	Median (IQR)	5.1 (0–13.6)
Pack-years	7987	Mean (SD)	11.8 (17.5)
Consumed tobacco (grams/day)	8520	Mean (SD)	3.0 (8.1)
Physical activity^*c*^	8767	–	–
Inactive	1877	%	21.4
Low	3989	%	45.5
Medium or high	2901	%	33.1
BMI (kg/m^2^)	8781	Median (IQR)	27.3 (22.8–30.1)
Categorized BMI (No., %)			
<25 kg/m^2^	2394	%	27.3
25-<30 kg/m^2^	4129	%	47.0
≥30 kg/m^2^	2258	%	25.7
Fruit consumption	8498	–	–
<once/day	3245	%	38.2
Vegetable consumption	8560	–	–
<once/day	5525	%	64.5
Meat consumption	8521	–	–
≥once/day	2785	%	32.7
Total cholesterol (mg/dL)	8771	Median (IQR)	221 (188–252)
CRP (mg/L)	8665	Median (IQR)	2.1 (1.0–4.5)
OxGua (μg/g creatinine)	8793	Median (IQR)	146 (107–203)

Abbreviations: BMI, body mass index; CRP, C-reactive protein; IQR, interquartile range; OxGua: oxidized guanine/guanosine, including 8-hydroxyguanine (8-OHGua) and its nucleoside forms 8-hydroxy-2′-deoxyguanosine (8-OHdGuo) and 8-hydroxyguanosine (8-OHGuo).

^a^*n* does not always add up to the total (*n* = 8,793) because of missing values.

^b^The alcohol consumption was calculated by the following equation: 1 bottle of beer = 11.88 g alcohol, 1 glass of wine = 22.0 g alcohol, 1 shot of liquor = 6.4 g alcohol.

^c^“Inactive” was defined by doing <1 hour of vigorous or light physical activity per week. “Medium or high” was defined by doing ≥2 h/week of vigorous and ≥2 h/week of light physical activity. All other amounts of physical activity were grouped into the category “Low”.

No variable had more than 10% of missing values and these missing values were imputed by multiple imputation^24^. The imputation model consisted of total cholesterol, CRP and the variables of the main model. Five complete data sets were generated by multiple imputation and results of Cox regression were combined by using the MIANALYZE procedure of the SAS software.

## Results

Table 1 shows that the mean age of the analyzed study population was 62 years and more females (55.2%) were included than males. Table 2 presents that the OxGua levels were positively, statistically significantly associated with female sex, low education, physical inactivity, and higher CRP levels, whereas a negative direction of the association was observed with alcohol consumption. However, OxGua levels were not associated with age and total cholesterol to a relevant extent, and associations with smoking, BMI and dietary factors were weak or not observed.

**Table 2:** Baseline characteristics of total study participants across tertiles of OxGua concentration (μg/g creatinine), ESTHER Study (2000–2016).

Characteristics	Tertile 1 (≤119.6)		Tertile 2 (>119.6–≤180.2)		Tertile 3 (>180.2)	
	*n* ^a^	Values	*n* ^a^	Values	*n* ^a^	Values
**Age (years, median, IQR)**	2931	63 (57–66)	2931	62 (57–67)	2931	62 (57–67)
**Sex (%)**	2931		2931		2931	
Female	1179	40.2	1672	**57.1**	2002	**68.3**
**Education (%)**	2857		2865		2846	
<9 years	2060	72.1	2150	**75.0**	2185	**76.8**
9–11 years	423	14.9	412	**14.4**	392	**13.8**
≥12 years	372	13.0	303	**10.6**	269	**9.4**
**Smoking status (%)**	2843		2928		2834	
Never smoker	1326	46.4	1413	**49.9**	1550	**54.8**
Former smoker, quitted >20 years	490	17.2	419	**14.8**	344	**12.2**
Former smoker, quitted 5–≤20 years	426	14.9	397	**14.0**	334	**11.8**
Former smoker, quitted 0–≤5 years	131	4.6	132	**4.7**	120	**4.2**
Current smoker, 0–≤15 g tobacco/day	203	7.1	202	**7.1**	215	**7.6**
Current smoker, 15 – ≤30 g tobacco/day	237	8.3	238	**8.4**	241	**8.5**
Current smoker, >30 g tobacco/day	44	1.5	33	**1.2**	25	**0.9**
**Consumed tobacco (grams/day, mean, SD)**	2857	3.1 (8.4)	2834	3.0 (8.1)	2829	3.0 (7.8)
**Pack-years (mean, SD)**	2675	13.1 (18.4)	2666	**11.7 (17.2)**	2646	**10.5 (16.8)**
**Alcohol consumption (g/day, median, IQR)**	2660	6.3 (0–16.1)	2655	**5.2 (0–13.5)**	2608	**3.4 (0–12.0)**
**Physical activity (%)**	2923		2923		2921	
Inactive	549	18.8	634	**21.7**	694	**23.8**
Low	1313	44.9	1339	**45.8**	1337	**45.8**
Medium or high	1061	36.3	950	**32.5**	890	**30.5**
**BMI (kg/m** ^**2**^ **, median, IQR)**	2928	27.3 (24.9–30.0)	2925	27.2 (24.7–30.1)	2928	27.3 (24.7–30.1)
**Categorized BMI (%)**						
<25 kg/m^2^	759	25.9	826	28.2	809	27.6
25- <30 kg/m^2^	1424	48.6	1351	46.2	1354	46.2
≥30 kg/m^2^	745	25.4	748	25.6	765	26.1
**Fruit consumption**	2850		2827		2821	
<once/day (%)	1168	41.0	1079	**38.2**	998	**35.4**
**Vegetable consumption**	2862		2846		2852	
<once/day (%)	1895	66.2	1875	65.9	1755	**61.5**
**Meat consumption**	2854		2836		2831	
≥once/day (%)	969	34.0	908	32.0	908	32.1
**Total cholesterol (mg/dL, median, IQR)**	2909	219 (184–249)	3023	**223 (189–253)**	2926	**222 (188–253)**
**CRP (mg/L, median, IQR)**	2886	2.0 (1.0–4.1)	2884	**2.1 (1.0–4.3)**	2895	**2.2 (1.0–5.0)**

Abbreviations: BMI, body mass index; CRP, C-reactive protein; IQR, interquartile range; OxGua, oxidized guanine/guanosine, including 8-hydroxyguanine (8-OHGua) and its nucleoside forms 8-hydroxy-2′-deoxyguanosine (8-OHdGuo) and 8-hydroxyguanosine (8-OHGuo); SD, standard deviation.

Note: Numbers in bold: Statistically significantly different from the bottom tertile (P < 0.05; chi-square test for categorical variables and Wilcoxon-Mann-Whitney test for continuous variables).

^a^*n* does not always add up to the total (*n* = 8,793) because of missing values.

During 14 years of follow-up, 1,540 participants were diagnosed with incident cancer, including 207 lung cancers, 196 colorectal cancers, 221 breast cancers and 246 prostate cancers. Table 3 shows the associations of OxGua levels with overall and site-specific cancer incidences in the total population and for the age groups 50–64 years and 65–74 years. No statistically significant associations were observed. In addition, no statistically significant findings were observed in the other subgroup analyses after correcting for multiple testing with either the FDR or Bonferroni correction. In total, we carried out 41 tests for potential associations of OxGua levels and the 5 cancer outcomes in the total population and in subgroup analyses defined by age, sex, smoking and obesity. Therefore, a Bonferroni-corrected p-value < 0.0012 would have been needed for a statistically significant finding. The lowest observed p-value in all analyses was 0.006 (for 1 SD increase in OxGua levels and total cancer incidence in current non-smokers), which is above the Bonferroni corrected p-value for statistical significance. The same result was obtained with FDR correction, which led to a statistically non-significant p-value of 0.162 for the strongest association observed in all analyses.

**Table 3:** Association of OxGua levels with overall and common site-specific cancer incidences in analyses in the total population and stratified by age, the ESTHER Study (2000–2016).

Cancer sites	OxGua levels [μg/g creatinine]	Total population			50–64 years			65–74 years		
		*n*_cases_/*n*_participants_	HR (95% CI)^a^	p-value	*n*_cases_/*n*_participants_	HR (95% CI)^a^	p-value	*n*_cases_/*n*_participants_	HR (95% CI)^a^	p-value
Overall										
Tertile 1	≤120	525/2931	ref.	—	293/1907	ref.	—	232/1025	ref.	—
Tertile 2	120–180	507/2931	1.02 (0.90, 1.15)	0.796	288/1837	1.06 (0.90, 1.25)	0.382	219/1092	0.96 (0.79, 1.16)	0.658
Tertile 3	>180	508/2931	1.06 (0.93, 1.21)	0.357	280/1785	1.09 (0.92, 1.30)	0.294	228/1147	1.02 (0.84, 1.24)	0.824
Increase per 1 SD		1540/8793	1.03 (0.99, 1.06)	0.163	861/5529	1.01 (0.96, 1.05)	0.696	679/3264	1.06 (0.98, 1.15)	0.116
Lung										
Tertile 1	≤120	69/2931	ref.	—	38/1907	ref.	—	31/1025	ref.	—
Tertile 2	120–180	75/2931	1.25 (0.89, 1.75)	0.244	48/1837	1.43 (0.92, 2.22)	0.109	27/1092	0.95 (0.55, 1.62)	0.820
Tertile 3	>180	63/2931	1.16 (0.81, 1.65)	0.419	39/1785	1.30 (0.81, 2.08)	0.268	24/1147	1.02 (0.58, 1.78)	0.943
Increase per 1 SD		207/8793	0.96 (0.85, 1.08)	0.494	125/5529	0.91 (0.77, 1.08)	0.229	82/3264	1.07 (0.86, 1.34)	0.576
Colorectal										
Tertile 1	≤120	63/2931	ref.	—	31/1907	ref.		32/1025	ref.	—
Tertile 2	120–180	72/2931	1.22 (0.87, 1.72)	0.230	34/1837	1.25 (0.77, 2.03)	0.338	38/1092	1.23 (0.76, 2.00)	0.405
Tertile 3	>180	61/2931	1.12 (0.78, 1.61)	0.537	34/1785	1.36 (0.84, 2.24)	0.224	27/1147	0.93 (0.54, 1.59)	0.788
Increase per 1 SD		196/8793	1.06 (0.99, 1.14)	0.095	99/5529	1.05 (0.99, 1.12)	0.153	97/3264	1.08 (0.88, 1.33)	0.480
Breast^b^										
Tertile 1	≤134	79/1603	ref.	—	53/1045	ref.	—	26/557	ref.	—
Tertile 2	134–200	68/1633	0.84 (0.61, 1.17)	0.701	45/1027	0.86 (0.58, 1.28)	0.537	23/606	0.81 (0.46, 1.42)	0.859
Tertile 3	>200	71/1617	0.89 (0.64, 1.23)	0.565	47/974	0.93 (0.62, 1.39)	0.704	24/644	0.79 (0.45, 1.38)	0.597
Increase per 1 SD		218/4853	0.95 (0.82, 1.11)	0.513	145/3046	0.95 (0.80, 1.13)	0.557	73/1807	0.93 (0.68, 1.28)	0.648
Prostate^c^										
Tertile 1	≤106	86/1314	ref.	—	51/867	ref.	—	35/448	ref.	—
Tertile 2	106–157	80/1312	0.91 (0.67, 1.24)	0.596	36/813	0.74 (0.48, 1.13)	0.815	44/498	1.14 (0.73, 1.80)	0.770
Tertile 3	>157	79/1314	0.89 (0.66, 1.22)	0.571	49/803	1.04 (0.71, 1.55)	0.461	30/511	0.74 (0.45, 1.21)	0.094
Increase per 1 SD		245/3940	1.05 (0.96, 1.16)	0.240	136/2483	1.06 (0.98, 1.16)	0.357	109/1457	1.05 (0.77, 1.45)	0.728

Abbreviations: CI, confidence interval; HR, hazard ratio; *n*, number of participants; OxGua, oxidized guanine/guanosine, including 8-hydroxyguanine (8-OHGua) and its nucleoside forms 8-hydroxy-2′-deoxyguanosine (8-OHdGuo) and 8-hydroxyguanosine (8-OHGuo); SD, standard deviation.

Note: Numbers in bold: statistically significant estimate compared to the bottom tertile (*P* < 0.05).

^a^The main model is adjusted for sex, smoking status, alcohol consumption, physical activity, body mass index and dietary factors (fruit, vegetable and red meat consumption).

^b^Only accessed in female participants and therefore model is not adjusted for sex.

^c^Only accessed in male participants and therefore model is not adjusted for sex.

Therefore, the following observed associations with p-values < 0.05 (before correction for multiple testing was performed) are not statistically significant and shall only be regarded to be hypotheses generating. In analyses stratified by sex, an association between OxGua levels and colorectal cancer was observe among women (HR (95% CI) per 1 SD increase: 1.09 (1.02, 1.17)) (Table 4). Stratified by smoking status, a 1 SD increase in OxGua levels was associated with a 5% increase in total cancer incidence (HR (95% CI): 1.05 (1.02, 1.10)), an 8% increase in colorectal cancer (HR (95% CI): 1.08 (1.01, 1.16)) and a 13% increase in prostate cancer incidence (HR (95% CI): 1.13 (1.01, 1.28)) among current non-smokers (Table 5). In current smokers, however, OxGua levels were negatively associated with prostate cancer risk (for comparison of top and bottom tertile) (Table 5). Splitting up the current non-smokers into never and former smokers did not reveal differential results (data not shown). Subgroup analyses in non-obese and obese study participants are shown in Table 6. The only finding was for colorectal cancer among non-obese individuals (HR (95% CI) per 1 SD increase: 1.08 (1.00, 1.16)).

**Table 4:** Association of OxGua levels with total and common site specific cancer incidences stratified by sex, the ESTHER Study (2000–2016).

Cancern sites	Women				Men			
	OxGua levels [μg/g creatinine]	*n*_cases_/*n*_participants_	HR (95% CI)^a^	p-value	OxGua levels [μg/g creatinine]	*n*_cases_/*n*_participants_	HR (95% CI)^a^	p-value
Overall								
Tertile 1	≤134	231/1603	ref.	—	≤106	273/1314	ref.	—
Tertile 2	134–200	233/1633	0.97 (0.81, 1.16)	0.718	106–157	279/1312	1.01 (0.85, 1.19)	0.883
Tertile 3	>200	241/1617	1.01 (0.84, 1.21)	0.531	>157	283/1314	1.00 (0.85, 1.19)	0.445
Increase per 1 SD		705/4853	1.00 (0.95, 1.05)	0.899		835/3939	1.06 (1.00, 1.13)	0.065
Lung								
Tertile 1	≤134	15/1603	ref.	—	≤106	43/1314	ref.	—
Tertile 2	134–200	25/1633	1.49 (0.79, 2.84)	0.648	106–157	46/1312	1.02 (0.67, 1.56)	0.278
Tertile 3	>200	21/1617	1.25 (0.65, 2.41)	0.566	>157	57/1314	1.21 (0.80, 1.82)	0.584
Increase per 1 SD		61/4853	0.91 (0.72, 1.16)	0.462		146/3939	0.98 (0.86, 1.11)	0.724
Colorectal								
Tertile 1	≤134	24/1603	ref.	—	≤106	34/1314	ref.	—
Tertile 2	134–200	26/1633	1.04 (0.60, 1.82)	0.689	106–157	46/1312	1.33 (0.85, 2.08)	0.185
Tertile 3	>200	28/1617	1.11 (0.64, 1.91)	0.661	>157	38/1314	1.09 (0.68, 1.73)	0.775
Increase per 1 SD		78/4853	**1.09 (1.02, 1.17)**	**0.008**		118/3939	0.95 (0.75, 1.21)	0.677

Abbreviations: CI, confidence interval; HR, hazard ratio; *n*, number of participants; OxGua, oxidized guanine/guanosine, including 8-hydroxyguanine (8-OHGua) and its nucleoside forms 8-hydroxy-2′-deoxyguanosine (8-OHdGuo) and 8-hydroxyguanosine (8-OHGuo); SD, standard deviation.

Note: Numbers in bold: statistically significant estimate compared to the bottom tertile (*P* < 0.05).

^a^The main model is adjusted for age, physical activity, body mass index, detailed smoking status, alcohol consumption and dietary factors (fruit, vegetable and red meat consumption).

**Table 5:** Association of OxGua levels with total and common site specific cancer incidences in analyses stratified by current smoking status, the ESTHER Study (2000–2016).

Cancer sites	OxGua levels [μg/g creatinine]	Current non-smokers			Current smokers		
		*n*_cases_/*n*_participants_	HR (95% CI)^a^	p-value	*n*_cases_/*n*_participants_	HR (95% CI)^a^	p-value
Overall							
Tertile 1	≤120	396/2416	ref.	—	129/515	ref.	—
Tertile 2	120–180	393/2433	1.03 (0.89, 1.18)	0.692	114/498	0.97 (0.74, 1.25)	0.794
Tertile 3	>180	415/2423	1.14 (0.99, 1.32)	0.077	93/508	0.82 (0.62, 1.09)	0.179
Increase per 1 SD		1204/7272	**1.05 (1.02, 1.10)**	**0.006**	336/1521	0.89 (0.67, 1.17)	0.389
Lung							
Tertile 1	≤120	30/2416	ref.	—	39/515	ref.	—
Tertile 2	120–180	38/2433	1.48 (0.91, 2.41)	0.114	37/498	1.08 (0.68, 1.73)	0.836
Tertile 3	>180	26/2423	1.19 (0.69, 2.07)	0.524	37/508	1.10 (0.69, 1.77)	0.680
Increase per 1 SD		94/7272	1.00 (0.86, 1.16)	0.969	113/1521	0.92 (0.75, 1.08)	0.462
Colorectal							
Tertile 1	≤120	54/2416	ref.	—	9/515	ref.	—
Tertile 2	120–180	62/2433	1.22 (0.85, 1.77)	0.263	10/498	1.16 (0.44, 3.04)	0.781
Tertile 3	>180	56/2423	1.20 (0.81, 1.76)	0.349	5/508	0.61 (0.18, 2.03)	0.411
Increase per 1 SD		172/7272	**1.08 (1.01, 1.16)**	**0.026**	24/1521	0.85 (0.39, 1.84)	0.680
Breast^b^							
Tertile 1	≤134	66/1380	ref.	—	13/223	ref.	—
Tertile 2	134–200	59/1379	0.91 (0.64, 1.29)	0.567	9/254	0.61 (0.25, 1.48)	0.606
Tertile 3	>200	60/1360	0.92 (0.65, 1.32)	0.151	11/257	0.71 (0.31, 1.64)	0.638
Increase per 1 SD		185/4119	0.99 (0.88, 1.12)	0.285	33/734	0.44 (0.18, 1.07)	0.073
Prostate^c^							
Tertile 1	≤106	63/1051	ref.	—	23/263	ref.	—
Tertile 2	106–157	62/1056	0.97 (0.68, 1.38)	0.828	18/256	0.77 (0.40, 1.49)	0.138
Tertile 3	>157	70/1046	1.06 (0.74, 1.50)	0.791	9/268	**0.41 (0.18, 0.96)**	**0.031**
Increase per 1 SD		195/3153	**1.13 (1.01, 1.28)**	**0.037**	50/787	0.48 (0.15, 1.51)	0.212

Abbreviations: CI, confidence interval; HR, hazard ratio; *n*, number of participants; OxGua, oxidized guanine/guanosine, including 8-hydroxyguanine (8-OHGua) and its nucleoside forms 8-hydroxy-2′-deoxyguanosine (8-OHdGuo) and 8-hydroxyguanosine (8-OHGuo); SD, standard deviation.

Note: Numbers in bold: statistically significant estimate compared to the bottom tertile (*P* < 0.05).

^a^The main model is adjusted for age, sex, physical activity, body mass index, detailed smoking status, alcohol consumption and dietary factors (fruit, vegetable and red meat consumption).

^b^Only accessed in female participants and therefore model is not adjusted for sex.

^c^Only accessed in male participants and therefore model is not adjusted for sex.

**Table 6:** Association of OxGua levels with total and common site specific cancer incidences in analyses stratified by obesity (BMI <30/≥30 kg/m^2^), the ESTHER Study (2000–2016).

Cancer sites	OxGua levels [μg/g creatinine]	No obesity			Obesity		
		*n*_cases_/*n*_participants_	HR (95% CI)^a^	p-value	*n*_cases_/*n*_participants_	HR (95% CI)^a^	p-value
**Overall**							
Tertile 1	≤120	392/2186	ref.	—	133/747	ref.	—
Tertile 2	120–180	370/2179	1.01 (0.88, 1.17)	0.860	137/750	1.04 (0.81, 1.32)	0.818
Tertile 3	>180	358/2163	1.03 (0.89, 1.20)	0.670	150/768	1.15 (0.90, 1.47)	0.292
Increase per 1 SD		1120/6528	1.03 (0.99, 1.07)	0.182	420/2265	1.03 (0.95, 1.11)	0.480
**Lung**							
Tertile 1	≤120	49/2186	ref.	—	20/747	ref.	—
Tertile 2	120–180	56/2179	1.30 (0.87, 1.94)	0.215	19/750	1.07 (0.57, 1.99)	0.856
Tertile 3	>180	48/2163	1.26 (0.83, 1.90)	0.329	15/768	0.88 (0.43, 1.80)	0.712
Increase per 1 SD		153/6528	0.99 (0.90, 1.09)	0.698	54/2265	0.82 (0.53, 1.29)	0.390
**Colorectal**							
Tertile 1	≤120	45/2186	ref.	—	18/747	ref.	—
Tertile 2	120–180	56/2179	1.36 (0.91, 2.02)	0.129	16/750	0.92 (0.47, 1.82)	0.760
Tertile 3	>180	41/2163	1.08 (0.70, 1.68)	0.730	20/768	1.23 (0.64, 2.39)	0.626
Increase per 1 SD		142/6528	**1**.**08** (**1**.**00**, **1**.**16**)	**0**.**043**	54/2265	0.99 (0.76, 1.30)	0.942
**Breast** ^**b**^							
Tertile 1	≤134	57/1166	ref.	—	22/436	ref.	—
Tertile 2	134–200	50/1219	0.84 (0.57, 1.22)	0.881	18/415	0.91 (0.48, 1.73)	0.356
Tertile 3	>200	49/1180	0.85 (0.58, 1.25)	0.653	22/437	1.00 (0.54, 1.84)	0.715
Increase per 1 SD		156/3565	0.92 (0.73, 1.15)	0.426	62/1288	1.00 (0.83, 1.21)	0.958
**Prostate** ^**c**^							
Tertile 1	≤106	70/983	ref.	—	16//331	ref.	—
Tertile 2	106–157	63/992	0.88 (0.63, 1.24)	0.455	17/320	1.07 (0.53, 2.16)	0.765
Tertile 3	>157	63/988	0.88 (0.62, 1.24)	0.787	6/326	0.97 (0.48, 1.94)	0.472
Increase per 1 SD		196/2963	1.06 (0.97, 1.17)	0.207	49/977	1.04 (0.74, 1.48)	0.775

Abbreviations: CI, confidence interval; HR, hazard ratio; *n*, number of participants; OxGua, oxidized guanine/guanosine, including 8-hydroxyguanine (8-OHGua) and its nucleoside forms 8-hydroxy-2′-deoxyguanosine (8-OHdGuo) and 8-hydroxyguanosine (8-OHGuo); SD, standard deviation.

Note: Numbers in bold: statistically significant estimate compared to the bottom tertile (*P* < 0.05).

^a^The main model is adjusted for age, sex, detailed smoking status, physical activity, alcohol consumption and dietary factors (fruit, vegetable and red meat consumption).

^b^Only accessed in female participants and therefore model is not adjusted for sex.

^c^Only accessed in male participants and therefore model is not adjusted for sex.

In a sensitivity analysis, modelling smoking with the variables “current average amount of consumed grams of tobacco per day” or “pack-years of smoking” did not change the results (data not shown). In a further sensitivity analysis, no relevant changes of the findings were observed after excluding cancer cases during the first 2 years of follow-up (data not shown).

## Discussion

In this large cohort of older adults, OxGua levels were not associated with any cancer outcome in the total population. Although not statistically significant after correction for multiple testing, some potential associations (raw p-values < 0.05) between OxGua levels and cancer incidences were observed in subgroup analyses. Positive associations were observed between OxGua levels and colorectal cancer among current non-smokers, women and non-obese participants. In addition, OxGua levels were positively associated with overall and prostate cancer incidence among current non-smokers. In contrast, OxGua levels were inversely associated with prostate cancer incidence among current smokers.

The OxGua molecules are derived from repair products of the oxidatively generated DNA/RNA lesions^12^. The formation of OxGua is not organ-specific and its excretion into urine is reflecting the average production in all parts of the body. Therefore, a statistically significant association with total cancer incidence was expected which would suggest that OS-induced genome instability may be involved in the etiology of various types of cancer among^25^. However, a potential association of OxGua levels and total cancer incidence was only observed among non-smokers. A possible explanation might be that a weak association cannot be detected among current smokers because smoking is a much stronger cancer risk factor than oxidatively generated DNA/RNA damage and overshadows the latter by increasing the absolute cancer risk of smokers leading to very weak relative risks for other risk factors.

This statistical explanation, may also explain the findings for colorectal cancer. Oxidatively generated DNA/RNA damage was only detected as a risk factor for colorectal cancer in the absence of other strong risk factors for this cancer entity, such as smoking, obesity and male sex^26^.

It is more difficult to explain the different directions of the observed effect estimates for associations of OxGua levels with prostate cancer incidence among current smokers and current non-smokers. OxGua levels were positively associated with prostate cancer incidence in non-smokers and inversely associated with prostate cancer incidence in current smokers. Among current non-smokers, the positive direction of the association can be explained by the fact that OS activates androgen receptor signaling^27^, which can promote prostate cancer development^29^. Among current smokers, the protective role of OS in prostate cancer development is unexpected because other observational studies showed that cigarette smoking is a risk factor for prostate cancer and smoking is known to be associated with OS^28^. The potential mechanism might be the cytotoxicity of OS caused by cigarette smoke in prostate tissue. OS plays an important role in determining cell fate and the effect of OS on cells largely relies on its levels^30^. Therefore, higher levels of OS of smokers may lead to apoptosis of prostate cancer cells. However, this explanation is a speculation and it should be remembered that the observed protective association could also be a random finding because the association was not statistically significant after correction of multiple testing. Interestingly, another analysis in the ESTHER cohort with 8-isoprostane levels also detected an inverse association of the OS biomarker with prostate cancer among current smokers^31^. Therefore, this initially unexpected finding deserves further investigations in other studies.

There is no previous prospective epidemiological study that estimated the association of OxGua levels with the risk of colorectal or prostate cancer to which we could compare our results. With respect to breast cancer, while it was observed a borderline statistically significant association of urinary 8-OHdGuo with breast cancer in a general population based on a Danish nested case-control study^17^, a Chinese population based nested case-control study did not confirm it^19^.

Our findings are consistent with two Danish nested case-control studies, which did not observe an association of 8-OHGua and 8-OHdGuo levels with lung cancer incidence in the total population^15,16^. The authors only observed an increased lung cancer incidence rate ratio with increasing 8-OHdG levels in subgroups (men, never smoker and former smoker)^15,16^. Albeit not statistically significant, our study also observed an increased risk for lung cancer at high OxGua levels only among non-smokers but not smokers.

Strengths include the prospective design, the long term cancer registry based follow-up and the large sample size. Although residual confounding cannot be completely excluded, detailed adjustment for potential confounders limited the extent of confounding as far as possible. In addition, reverse causality bias was unlikely because excluding cancers from the first two years of follow-up did not change the results.

There are also limitations need to be considered when interpreting the results. First, OxGua levels were measured with single measurements because of limited funding. This may have affected the precision of measurements for single study participants but at the population level, the large sample size (n = 8,793) minimized the influence of random measurement errors on the associations with the outcomes. Second, ELISA assays have the general limitation of a lower specificity for the target molecule(s), when compared to mass spectrometry methods. As outlined in detail in the methods section, the chosen ELISAs also measure structurally related molecules, including biologically relevant metabolites. Of course, it would be even better to have distinct measurements of all these metabolites in order to assess their distinct associations with the outcomes (in particular for 8-OHGua from either DNA or RNA, 8-OHGuo from DNA and 8-OHdGuo from RNA), which could differ, as shown previously for DNA and RNA oxidation for mortality and cardiovascular disease risk in diabetes patients^32^. Future studies are required to target potential differences for DNA and RNA oxidation for cancer outcomes with more specific methods. Third, studies for others ethnicities and younger study participants are needed because our results can only be generalized for older Caucasians.

To conclude, this prospective cohort study observed no association of urinary OxGua levels with lung, colorectal, breast and prostate cancer in the total population. Higher urinary OxGua levels were potentially associated with an increased risk of colorectal cancer only among current non-smokers, women and non-obese participants. Urinary OxGua levels also showed a positive association with overall, prostate cancer incidence among current non-smokers and an inverse association with prostate cancer incidence among current smokers. However, none of these findings from subgroup analyses were statistically significant after correction for multiple testing and further studies are needed to corroborate our findings. However, we are confident that our results can be validated by others because there seemed to be pattern in our study results that oxidatively generated DNA/RNA damage could be a weak cancer risk factor (especially for colorectal cancer) in the absence of other strong risk factors like smoking, obesity and male sex.

## Data Availability

Requests for access to the data used for this investigation can be made by inquiry at the corresponding author.
